# Long-term quality of life in critically ill patients with acute kidney injury treated with renal replacement therapy: a matched cohort study

**DOI:** 10.1186/s13054-015-1004-8

**Published:** 2015-08-06

**Authors:** Sandra Oeyen, Wouter De Corte, Dominique Benoit, Lieven Annemans, Annemieke Dhondt, Raymond Vanholder, Johan Decruyenaere, Eric Hoste

**Affiliations:** Faculty of Medicine and Health Sciences, Ghent University, De Pintelaan 185, 9000 Ghent, Belgium; Department of Intensive Care, Ghent University Hospital, De Pintelaan 185, 9000 Ghent, Belgium; Department of Anaesthesia and Intensive Care Medicine, AZ Groeninge Hospital, President Kennedylaan 4, 8500 Courtray, Belgium; Research Foundation – Flanders (FWO), Brussels, Belgium; I-CHER, Faculty of Medicine and Health Sciences, Ghent University, De Pintelaan 185, 9000 Ghent, Belgium; Department of Nephrology, Ghent University Hospital, De Pintelaan 185, 9000 Ghent, Belgium

## Abstract

**Introduction:**

Acute kidney injury (AKI) is a common complication in intensive care unit (ICU) patients and is associated with increased morbidity and mortality. We compared long-term outcome and quality of life (QOL) in ICU patients with AKI treated with renal replacement therapy (RRT) with matched non-AKI-RRT patients.

**Methods:**

Over 1 year, consecutive adult ICU patients were included in a prospective cohort study. AKI-RRT patients alive at 1 year and 4 years were matched with non-AKI-RRT survivors from the same cohort in a 1:2 (1 year) and 1:1 (4 years) ratio based on gender, age, Acute Physiology and Chronic Health Evaluation II score, and admission category. QOL was assessed by the EuroQoL-5D and the Short Form-36 survey before ICU admission and at 3 months, 1 and 4 years after ICU discharge.

**Results:**

Of 1953 patients, 121 (6.2 %) had AKI-RRT. AKI-RRT hospital survivors (44.6 %; *N* = 54) had a 1-year and 4-year survival rate of 87.0 % (*N* = 47) and 64.8 % (*N* = 35), respectively. Forty-seven 1-year AKI-RRT patients were matched with 94 1-year non-AKI-RRT patients. Of 35 4-year survivors, three refused further cooperation, three were lost to follow-up, and one had no control. Finally, 28 4-year AKI-RRT patients were matched with 28 non-AKI-RRT patients. During ICU stay, 1-year and 4-year AKI-RRT patients had more organ dysfunction compared to their respective matches (Sequential Organ Failure Assessment scores 7 versus 5, *P* < 0.001, and 7 versus 4, *P* < 0.001). Long-term QOL was, however, comparable between both groups but lower than in the general population. QOL decreased at 3 months, improved after 1 and 4 years but remained under baseline level. One and 4 years after ICU discharge, 19.1 % and 28.6 % of AKI-RRT survivors remained RRT-dependent, respectively, and 81.8 % and 71 % of them were willing to undergo ICU admission again if needed.

**Conclusion:**

In long-term critically ill AKI-RRT survivors, QOL was comparable to matched long-term critically ill non-AKI-RRT survivors, but lower than in the general population. The majority of AKI-RRT patients wanted to be readmitted to the ICU when needed, despite a higher severity of illness compared to matched non-AKI-RRT patients, and despite the fact that one quarter had persistent dialysis dependency.

**Electronic supplementary material:**

The online version of this article (doi:10.1186/s13054-015-1004-8) contains supplementary material, which is available to authorized users.

## Introduction

Acute kidney injury (AKI) treated with renal replacement therapy (RRT) affects approximately 5–10 % of intensive care unit (ICU) patients [1]. These patients are amongst the most severely ill patients in the ICU, as illustrated by the 50 % in-hospital mortality [2–4]. AKI-RRT patients who survive may develop chronic kidney disease, including end-stage renal disease, and experience decreased long-term survival [4–8]. Therefore, to fully appreciate outcomes of critically ill AKI-RRT survivors, indices regarding long-term morbidity and quality of life (QOL) should also be taken into account [9, 10].

Major reductions in long-term QOL in critically ill patients are seen in severe acute respiratory distress syndrome, prolonged mechanical ventilation, severe sepsis, and after major trauma, all conditions frequently associated with AKI-RRT [11]. Data regarding QOL in AKI-RRT patients show that these patients have a decreased QOL compared to the general population but perceive QOL as good [12, 13]. However, these studies were either retrospective [14–17], evaluated QOL after a short term [12–15, 17–21], lacked baseline QOL assessment [12–15, 18, 22], or dated back more than a decade [14–16, 18, 23]. It is also unclear whether impairment in long-term QOL is the consequences of critical illness, AKI-RRT, pre-existing co-morbidities, or a combination of these.

The aim of the present study was to assess long-term outcomes and QOL of critically ill AKI-RRT patients at baseline, and at 3 months, 1 year and 4 years after ICU discharge and to compare QOL with a cohort of matched non-AKI-RRT patients [24].

## Methods

### Design, patients, and setting

The cohort described in this study is a subgroup of a prospective observational cohort. During one year (March 2008 to March 2009), all consecutively admitted adult patients at the 14-bed medical ICU (MICU), the 22-bed surgical ICU (SICU), and the 6-bed burns unit of the Ghent University Hospital, Belgium, were screened to study QOL and cost-effectiveness of intensive care [25]. Exclusion criteria were age <16 years and admission to the ICU after cardiac surgery. In case of multiple ICU admissions, only the first was considered.

In this study, only AKI-RRT patients of the larger cohort were included. Chronic hemodialysis patients were excluded. The attending critical care physician and consulting nephrologist assessed indication for RRT and modality.

To study the impact of RRT on long-term outcome and QOL, we performed a matched cohort study, according to the STROBE guidelines [26]. Included AKI-RRT patients alive at 1 year after hospital discharge were defined as exposed patients and individually matched with 1-year non-AKI-RRT survivors (defined as nonexposed patients) from the same cohort. Being a patient in the non-AKI-RRT group did not imply normal kidney function: it implied no treatment with RRT. To correct for possible bias, we excluded patients who needed RRT but who did not receive RRT due to therapeutic restrictions. Equally, AKI-RRT patients alive at time of this study (average 4 years later) were individually matched with 4-year non-AKI-RRT survivors. The exposed to nonexposed ratio was aimed at 1:2 to reduce risk of selection bias. When there were more than two nonexposed patients for an exposed patient, only the nonexposed patient with the best overall match was selected. If an exposed patient could only be properly matched to one nonexposed patient, we accepted matching in a 1:1 ratio for the respective cohort in order to avoid an imbalance of characteristics and to retain the best possible matching. Matching was based on gender, age (±5 years), Acute Physiology and Chronic Health Evaluation (APACHE) II score (±5), and admission category.

### Data collection and definitions

Variables collected within the first 24 hours of ICU admission included age, gender, body mass index, personal, proxy, and family practitioner contact data, living situation, activity of daily living, co-morbidity as measured by the Charlson co-morbidity index [27], hospitalization in the last 6 months, main reason for ICU admission, APACHE II score [28], Sequential Organ Failure Assessment (SOFA) score [29], need for mechanical ventilation, use of any vasopressors, and need for RRT. During ICU stay, SOFA scores, need for mechanical ventilation, vasopressors, RRT, and do-not-resuscitate codes were collected on a daily base. ICU length of stay (LOS), hospital LOS, and vital status at ICU and hospital discharge, and at 3 months, 1 year and 4 years following ICU discharge, were collected for each patient.

Values of serum creatinine for AKI-RRT patients were extracted from the STARRT database, which includes all relevant renal and RRT data of ICU patients with AKI-RRT treated in our hospital, and from laboratory data in control patients. The estimated glomerular filtration rate (eGFR) was calculated using the Chronic Kidney Disease Epidemiology Collaboration formula [30]. Renal recovery was defined as independence from RRT.

The study was approved by the local ethical committee (Ethisch Comité Ghent University Hospital; amendment project 2007/423 approved 19 February 2013; B67020072805), and conducted in accordance with the declaration of Helsinki. A signed informed consent was obtained from every included patient.

### Quality of life

QOL was assessed by means of the Medical Outcomes Study 36-item Short Form Health Survey (SF-36v2®) and the EuroQoL-5D (EQ-5D). The SF-36 questionnaire contains 36 items measuring eight health domains: physical (PF) and social functioning (SF), role limitations due to physical (RP) or emotional problems (RE), mental health (MH), vitality (VT), bodily pain (BP), and general perception of health (GH) [31]. Two component scores, a physical (PCS) and a mental (MCS), are calculated summary scores where, respectively, the physical domains (PF, RP, BP, GH) or the mental domains (VT, SF, RE, MH) will account more in the score. We assessed SF-36 as norm-based scores to be able to compare them directly with the general healthy population, with a group level range of 47–53 considered as average or normal [31]. Group scores less than 47 indicate impaired functioning within that health domain; group scores greater than or equal to 53 should be considered average or above the normative sample.

The 36th item, health transition, provides information about perceived changes in health status. The validity and reliability of the SF-36 has been confirmed in critically ill patients, and its use is validated in face-to-face interviews, telephone interview, and questionnaire by regular mail [32].

The EQ-5D is a generic QOL questionnaire that measures health in five dimensions: mobility, self-care, usual activities, pain/discomfort, and anxiety/depression [33]. Each dimension has three levels: no problems, moderate problems, or severe problems. On a visual analogue scale (VAS), patients can rate their perceived overall health between 0 and 100. The EQ-5D is suitable for measuring QOL in critical care [34, 35].

QOL was assessed at different time points: baseline QOL and strictly at 3 months and 1 year after ICU discharge. QOL was also assessed in August 2013, a median of 4.1 years (3.9–4.3 years) after ICU discharge. Following ICU admission and study inclusion, a face-to-face interview to assess baseline QOL (defined as QOL 2 weeks before ICU admission) was performed as soon as possible. This interview was preferably taken from the patient, or when impossible, from the proxy. Three months, 1 year, and 4 years after ICU discharge, patients were sent the EQ-5D and SF-36 surveys by regular mail; at 1 and 4 years, questions concerning living situation, memories, sleep quality, and willingness to be readmitted to an ICU department were added. If the questionnaires were not returned within 1 month, patients or relatives were contacted by phone to assess QOL after 1 year and after 4 years. Eventually, the family practitioner was contacted.

### Statistical analysis

Data are expressed as median (interquartile range; IQR) for continuous variables and as number (%) for categorical variables. QOL at the different time points and characteristics between both groups (AKI-RRT versus non-AKI-RRT patients) were compared by the Mann–Whitney *U* test for continuous variables and by the Chi-square test for categorical variables. For long-term analysis of QOL, differences between QOL at baseline (only hospital survivors), at 3 months and at 1 and 4 years after ICU discharge were assessed by Chi-square (EQ-5D) or Friedman test (SF-36). *P* values were two-sided and statistical significance was set at 0.05. All statistical analyses were done using IBM SPSS Statistics software version 21 (IBM, Armonk, New York, USA).

## Results

### Characteristics of the study population

**Fig. 1:**
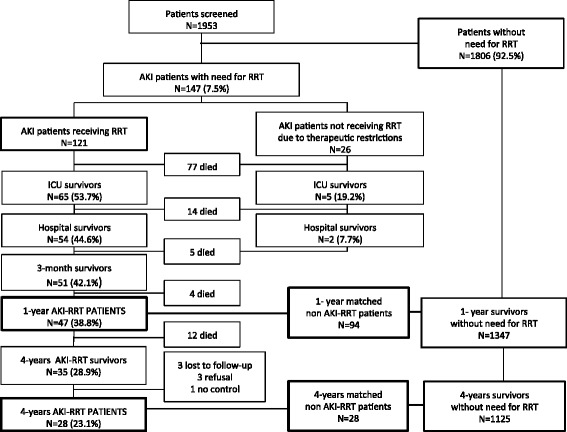
Patient cohort. *N* number, *AKI* acute kidney injury, *RRT* renal replacement therapy, *ICU* intensive care unit

During the 1-year study period 1953 patients were included (Fig. 1). Of these, 147 patients (7.5 %) developed AKI with need for RRT. Of these, 121 patients (6.2 %) received RRT. ICU (46.3 %), hospital (55.4 %), 3-month (57.9 %), 1-year (61.1 %) and 4-year (71.1 %) mortality rates in these patients were high. Twenty-six AKI patients (1.3 %) did not receive RRT due to therapeutic restrictions and were excluded from further analysis.

AKI-RRT hospital survivors (44.6 %) had a 1-year and 4-year survival rate of 87.0 % and 64.8 %, respectively. Forty-seven 1-year AKI-RRT survivors were individually matched with 94 1-year non-AKI-RRT survivors (two matches for all AKI-RRT patients). Of 35 4-year survivors, three refused further cooperation, three were lost-to-follow-up, and one had a double match. In 13 of the 28 included 4-year AKI-RRT survivors, only one good match could be found, so matching occurred in a 1:1 ratio. Finally, 28 4-year AKI-RRT survivors were individually matched with 28 non-AKI-RRT patients. AKI-RRT and non-AKI-RRT patients had similar gender, age, APACHE II score, and admission category at 1 year and 4 years (Table 1).

**Table 1:** Patient characteristics at ICU admission, organ failure during ICU admission, and outcomes

	1-year AKI-RRT patients	1-year non-AKI-RRT patients	*P*	4-year AKI-RRT patients	4-year non-AKI-RRT patients	*P*
	(*N* = 47)	(*N* = 94)		(*N* = 28)	(*N* = 28)	
Age (years)	57 (45–69)	57 (48–70)	0.897	54 (45–66)	53 (45–68)	0.718
Male gender	31 (66.0)	62 (66.0)	0.999	16 (57.1)	16 (57.1)	0.999
Body mass index (kg/m^2^)	26.2 (22.8-29.7)	25.9 (22.0-29.4)	0.444	27.3 (22.9-31.6)	24.5 (22.9-27.8)	0.092
Serum creatinine baseline (mg/dL)^a^	1.14 (0.94-1.51)	0.82 (0.66-1.04)	0.001	0.97 (0.80-1.26)	0.78 (0.65-1.11)	0.062
eGFR baseline (mL/min per 1.73 m^2^)^a^	86 (71–100)	100 (83–116)	0.007	99 (85–109)	102 (87–116)	0.629
Lives at home before admission	45 (95.7)	90 (95.75)	0.999	26 (92.9)	27 (96.4)	0.553
Activity of daily living						
No limitations	25 (53.2)	47 (50.0)	0.721	18 (63.4)	21 (75.0)	0.383
Moderate limitations	19 (40.4)	42 (44.7)	0.631	7 (25.0)	7 (25.0)	0.999
Chair-bound	0 (0)	3 (3.2)	0.216	0 (0)	0 (0)	NA
Bedridden	3 (6.4)	2 (2.1)	0.198	3 (10.7)	0 (0)	<0.001
Hospitalization in last 6 months before ICU	20 (42.6)	46 (48.9)	0.474	10 (35.7)	14 (50.0)	0.280
Charlson comorbidity index	1 (0–3)	2 (0–3)	0.115	0 (0–2)	2 (0–3)	0.110
Type of admission						
Medical	32 (68.1)	67 (71.3)	0.696	18 (64.3)	18 (64.3)	0.999
Scheduled surgery	1 (2.1)	4 (4.3)	0.519	0 (0)	4 (14.3)	0.038
Emergency surgery	10 (21.3)	18 (19.1)	0.765	7 (25.0)	3 (10.7)	0.163
Trauma	3 (6.4)	4 (4.3)	0.376	2 (7.1)	2 (7.1)	0.999
Burns	1 (2.1)	1 (1.1)	0.614	1 (3.6)	1 (3.6)	0.999
Severity of illness at ICU admission (first 24 hours)						
APACHE II score	26 (21–31)	24 (20–30)	0.251	23 (20–28)	22 (18–25)	0.362
SOFA score	9 (5–11)	7 (5–10)	0.047	7 (4–12)	6 (4–9)	0.139
Mechanical ventilation	29 (61.7)	49 (52.1)	0.281	21 (75.0)	13 (46.4)	0.029
Vasopressors	21 (44.7)	37 (39.4)	0.545	11 (39.3)	9 (32.1)	0.577
RRT	11 (23.4)	0 (0)	<0.001	6 (21.4)	0 (0)	0.010
Organ failure during ICU stay						
Mechanical ventilation	39 (83.0)	50 (53.2)	<0.001	24 (85.7)	13 (46.4)	0.002
Length of mechanical ventilation (days)	16 (3–27)	1 (0–3)	<0.001	18 (4–31)	0 (0–7)	<0.001
Vasopressors	36 (76.6)	42 (44.7)	<0.001	21 (75.0)	10 (35.7)	0.003
Length of vasopressor therapy (days)	5 (1–8)	0 (0–3)	<0.001	3 (0–10)	0 (0–3)	0.002
RRT	47 (100)	0 (0)	<0.001	28 (100.0)	0 (0)	<0.001
Mean SOFA score	7 (6–9)	5 (4–7)	<0.001	7 (5–10)	4 (4–7)	<0.001
Outcomes						
ICU length of stay (days)	22 (11–42)	5 (3–9)	<0.001	24 (13–49)	7 (3–10)	<0.001
Readmissions	8 (17.0)	12 (12.8)	0.495	3 (10.7)	4 (14.3)	0.686
Hospital LOS, days	70 (30–100)	21 (13–44)	<0.001	62 (20–130)	19 (10–46)	0.003
Do-not-resuscitate decisions	4 (8.5)	3 (3.2)	0.170	2 (7.1)	1 (3.6)	0.312
Long-term mortality	12 (25.5)	20 (21.3)	0.570	NA	NA	NA
Need for RRT at hospital discharge	12 (25.5)	NA	NA	10 (35.7)	NA	NA
Need for RRT at 3 months	9 (19.1)	NA	NA	8 (28.6)	NA	NA
Need for RRT at 1 year	9 (19.1)	NA	NA	8 (28.6)	NA	NA
Need for RRT at 4 years	NA	NA	NA	8 (28.6)	NA	NA
Living situation after 1 year	46 answers	93 answers		27 answers	26 answers	
Independent without additional help	25 (54.3)	47 (50.5)	0.672	16 (59.3)	14 (53.8)	0.691
Independent with some help	12 (26.1)	22 (23.7)	0.754	6 (22.2)	6 (23.1)	0.941
Together with relatives (others than spouse)	6 (13.0)	14 (15.1)	0.751	3 (11.1)	4 (15.4)	0.646
Special care facility	3 (6.5)	5 (5.4)	0.786	2 (7.4)	1 (3.8)	0.575
Other	0 (0)	5 (5.4)	0.109	0 (0)	1 (3.8)	0.304
Living situation after 4 years	NA	NA	NA	27 answers	26 answers	
Independent without additional help	NA	NA	NA	18 (66.7)	14 (53.8)	0.340
Independent with some help	NA	NA	NA	5 (18.5)	6 (23.1)	0.682
Together with relatives (others than spouse)	NA	NA	NA	2 (7.4)	5 (19.2)	0.204
Special care facility	NA	NA	NA	2 (7.4)	1 (3.8)	0.575
Other	NA	NA	NA	0 (0)	0 (0)	0.999

Values are given as median (interquartile range) or number (%) as appropriate. ^a^ Serum creatinine at baseline was defined as serum creatinine 6 months before ICU admission. Values were missing in 27 of the 1-year AKI-RRT patients, in 14 of the 94 1-year non-AKI-RRT patients, in 21 of the 4-year AKI-RRT patients, and in 4 of the 4-year non-AKI-RRT patients. *AKI* acute kidney injury, *APACHE* Acute Physiology and Chronic Health Evaluation, *eGFR* estimated glomerular filtration rate, *ICU* intensive care unit, *NA* not applicable, *RRT* renal replacement therapy, *SOFA* Sequential Organ Failure Assessment

During ICU stay, 1-year and 4-year AKI-RRT patients had higher SOFA scores compared to their respective matches, and more needed mechanical ventilation or vasopressors for a longer time (Table 1).

### Renal characteristics and renal outcomes

One-year AKI-RRT patients had higher baseline serum creatinine concentrations and lower eGFR compared to their matches. These measurements did not significantly differ between 4-year AKI-RRT and non-AKI-RRT patients (Table 1).

Respectively, 12 1-year (25.5 %) and 10 4-year AKI-RRT patients (35.7 %) were RRT-dependent at hospital discharge. Nine (19.1 %) of the 1-year and 8 (28.6 %) of the 4-year AKI-RRT patients remained RRT dependent over time.

### Quality of life

An overview of the persons who rated QOL, how QOL was assessed, and the number of completed QOL surveys is given in Table 2. Most patients rated their own QOL at the different time points, except at baseline in 1-year AKI-RRT patients.

**Table 2:** Persons who rated QOL, assessment of QOL, number of completed QOL surveys

	Baseline			3 Months			1 Year			4 Years		
	AKI-RRT	non-AKI-RRT	*P*	AKI-RRT	non-AKI-RRT	*P*	AKI-RRT	non-AKI-RRT	*P*	AKI-RRT	Non-AKI-RRT	*P*
1-Year survivors	*N* = 47^a^	*N* = 94^a^		*N* = 34^b^	*N* = 71^b^		*N* = 46^c^	*N* = 94^d^				
Patient	14 (29.8)	57 (60.6)	0.001	25 (73.5)	57 (80.3)	0.434	33 (71.7)	65 (69.1)	0.753			
Partner	15 (31.9)	17 (18.1)	0.065	2 (5.9)	7 (9.9)	0.496	7 (15.2)	13 (13.8)	0.826			
Son/daughter	8 (17.0)	9 (9.6)	0.200	3 (8.8)	4 (5.6)	0.540	1 (2.2)	8 (8.5)	0.151			
Other family	4 (8.5)	5 (5.3)	0.465	0 (0)	0 (0)	0.999	1 (2.2)	2 (2.1)	0.986			
Others	6 (12.8)	6 (6.4)	0.200	4 (11.8)	4 (11.8)	0.268	4 (8.7)	6 (6.4)	0.618			
4-Year survivors	*N* = 28^a^	*N* = 27^a^		*N* = 21^b^	*N* = 23^b^		*N* = 27^e^	*N* = 26^f^		*N* = 28^g^	*N* = 28^h^	
Patient	8 (28.6)	18 (66.7)	0.005	17 (81.0)	17 (73.9)	0.578	22 (81.5)	22 (84.6)	0.761	24 (85.7)	21 (77.8)	0.313
Partner	7 (25.0)	4 (14.8)	0.345	1 (4.8)	3 (13.0)	0.340	3 (11.1)	3 (11.5)	0.961	1 (3.6)	2 (7.4)	0.553
Son/daughter	6 (21.4)	2 (7.4)	0.140	2 (9.5)	1 (4.3)	0.496	1 (3.7)	0 (0)	0.322	0 (0)	2 (7.4)	0.150
Other family	3 (10.7)	3 (11.1)	0.962	0 (0)	1 (4.3)	0.334	0 (0)	0 (0)	0.999	0 (0)	2 (7.4)	0.150
Others	4 (14.3)	0 (0)	0.041	1 (4.8)	1 (4.3)	0.947	1 (3.7)	1 (3.4)	0.978	3 (10.7)	1 (3.7)	0.299

All values are shown as number (%). ^a^All QOL surveys completed by face-to-face interviews; ^b^all QOL surveys completed by regular mail; ^c^46 QOL surveys completed, 32 by regular mail (69.6 %) and 14 by phone interview (30.4 %); ^d^94 QOL surveys completed, 67 by regular mail (71.3 %) and 27 by phone interview (28.7 %); ^e^27 QOL surveys completed, 18 by regular mail (66.7 %) and 9 by phone interview (33.3 %); ^f^26 QOL surveys completed, 19 by regular mail (73.1 %) and 7 by phone interview (26.9 %); ^g^28 QOL surveys completed, 14 by regular mail (50.0 %) and 14 by phone interview (50.0 %); ^h^28 QOL surveys completed, 20 by regular mail (71.4 %) and 8 by phone interview (28.6 %). *AKI* acute kidney injury, *QOL* quality of life, *RRT* renal replacement therapy

**Fig. 2:**
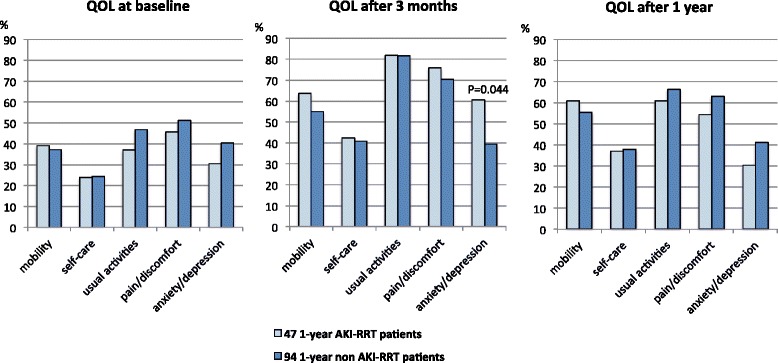
EQ-5D assessments in the 1-year cohort. Percentages of patients with some or severe problems per dimension at the three different time points. The X-axis represents the different dimensions of the EQ-5D. The Y-axis represents the percentages (%) of patients with some or severe problems in a respective dimension. Only significant *P* values (Chi-Square test) are shown above the respective dimensions. *AKI* acute kidney injury, *QOL* quality of life, *RRT* renal replacement therapy

**Fig. 3:**
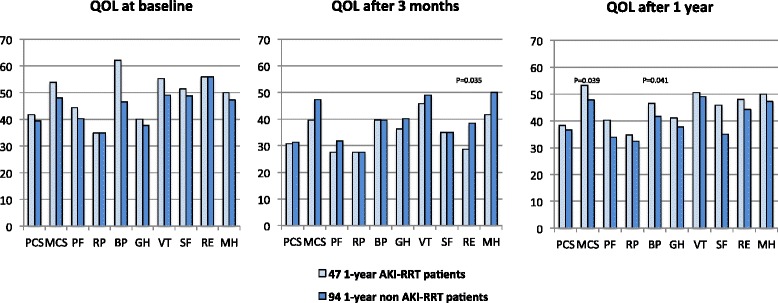
SF-36 assessments in the 1-year cohort. Norm-based median scores per domain at the three different time points. The X-axis represents the different domains of the SF-36. The Y-axis represents the norm-based median scores in a respective domain of the SF-36. A norm-based median score between 47 and 53 in a group of patients is considered as normal or average. Norm-based median scores below 47 indicate impaired functioning or below average; norm-based median scores above 53 indicate better functioning or above average. Only significant *P* values (Mann–Whitney U analysis) are shown above the respective domains. *AKI* acute kidney injury, *BP* bodily pain, *GH* general health, *MCS* mental component score, *MH* mental health, *PCS* physical component score, *PF* physical functioning, *QOL* quality of life, *RE* role emotional, *RP* role physical, *RRT* renal replacement therapy, *SF* social functioning, *VT* vitality

**Fig. 4:**
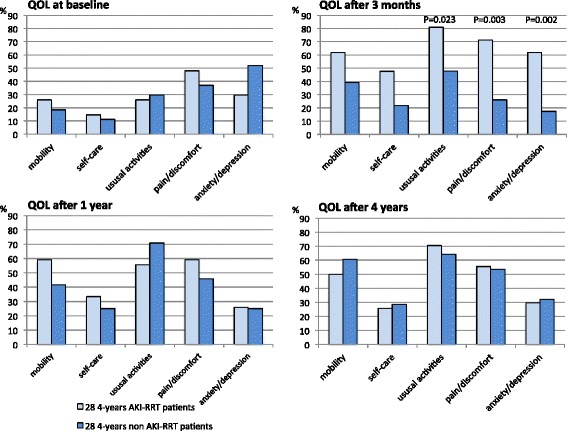
EQ-5D assessments in the 4-year cohort. Percentages of patients with some or severe problems per dimension at the four different time points. The X-axis represents the different dimensions of the EQ-5D. The Y-axis represents the percentages (%) of patients with some or severe problems in a respective dimension. Only significant *P* values (Chi Square test) are shown above the respective dimensions. *AKI* acute kidney injury, *QOL* quality of life, *RRT* renal replacement therapy

**Fig. 5:**
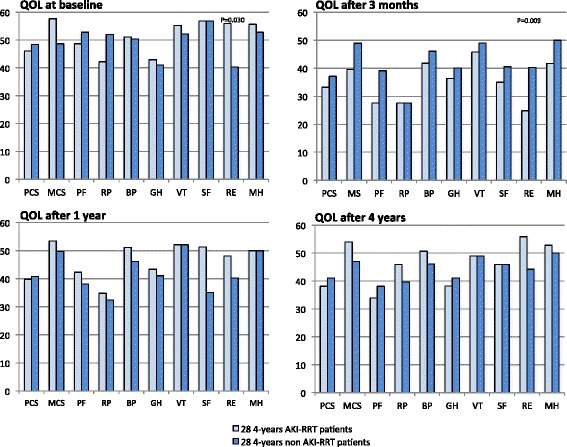
SF-36 assessments in the 4-year cohort. Norm-based median scores per domain at the four different time points. The X-axis represents the different domains of the SF-36. The Y-axis represents the norm-based median scores in a respective domain of the SF-36. A norm-based median score between 47 and 53 in a group of patients is considered as normal or average. Norm-based median scores below 47 indicate impaired functioning or below average; norm-based median scores above 53 indicate better functioning or above average. Only significant *P* values (Mann–Whitney U analysis) are shown above the respective domains. *AKI* acute kidney injury, *BP* bodily pain, *GH* general health, *MCS* mental component score, *MH* mental health, *PCS* physical component score, *PF* physical functioning, *QOL* quality of life, *RE* role emotional, *RP* role physical, *RRT* renal replacement therapy, *SF* social functioning, *VT* vitality

Significant differences in QOL between AKI-RRT and non-AKI-RRT survivors at each different time point were small. Figures 2 and 3 show that the 1-year AKI-RRT versus 1-year non-AKI-RRT patients had comparable baseline QOL. The 1-year AKI-RRT patients were poorer emotionally at 3 months (RE 28.7 versus 38.4; *P* = 0.035), but had a better mental score (MCS 53.3 versus 47.8; *P* = 0.039) and less bodily pain (BP 46.5 versus 41.6; *P* = 0.041) at 1 year (Fig. 3). Figures 4 and 5 show that the 4-year AKI-RRT versus 4-year non-AKI-RRT patients were emotionally better at baseline (RE 55.9 versus 40.3; *P* = 0.030) (Fig. 5), but had more problems with usual activities (81.0 % versus 47.8 %; *P* = 0.023), pain (71.4 % versus 26.1 %; *P* = 0.003) and anxiety (61.9 % versus 17.4 %; *P* = 0.002) at 3 months (Fig. 4). QOL after 1 and 4 years showed no differences (Figs. 4 and 5).

Comparing QOL within each group between the different time points revealed that QOL particularly decreased after 3 months.

### Evolution in QOL over time: 1-year cohort

All 1-year AKI-RRT patients reported more problems on the EQ-5D after 3 months compared to baseline. After 1 year, they experienced fewer problems but still more than before ICU admission. The EQ-5D showed the same evolution for 1-year non-AKI-RRT patients (Additional file 1A and B).

The SF-36 showed significant evolutions in QOL over time for 1-year AKI-RRT patients in nearly all dimensions. QOL decreased after 3 months and improved after 1 year, but without return to the baseline level. QOL also remained under the level of the average population. The same pattern, although less pronounced, was seen in 1-year non-AKI-RRT patients (Additional file 2A and B).

For 1-year AKI-RRT patients the median VAS scores ranged from 70 (baseline), to 60 (3 months) and 70 (1 year) (*P* = 0.048). In non-AKI-RRT patients the VAS remained the same: 68, 65 and 65 at baseline, 3 months and 1 year after ICU discharge, respectively (*P* = 0.917).

### Evolution in QOL over time: 4-year cohort

Changes in QOL over time assessed by the EQ-5D were significant in AKI-RRT patients for mobility (*P* = 0.040), usual activities (*P* < 0.001), and anxiety (*P* = 0.040) (Additional file 1C) and in 4-year non-AKI-RRT patients for mobility (*P =* 0.017), and usual activities (*P* = 0.014), with most problems at 3 months after ICU discharge followed by an improvement in QOL after 1 year (Additional file 1D). QOL never returned to baseline level.

The SF-36 showed that, in both groups, QOL decreased after 3 months compared to baseline (Additional file 2C and D). For the 4-year AKI-RRT patients, QOL improved after 1 year, especially in the mental domains. At 4 years, QOL significantly decreased physically but improved or remained the same in the mental components (Additional file 2C). Changes in long-term QOL in the 4-year non-AKI-RRT patients were less pronounced (Additional file 2D).

The 4-year AKI-RRT patients showed a decrease in VAS after 3 months (63), and improvements after 1 (70) and 4 years (68), but without regain of the baseline level (70) (*P* = 0.044). The 4-year non-AKI-RRT patients had the same evolution but without significance (*P* = 0.327).

Additional file 3 and Additional file 4 illustrate in more detail the variability in EQ-5D and SF-36 over time.

Overall, long-term QOL remained under the baseline level for AKI-RRT and non-AKI-RRT patients, and under the QOL of the average population.

### Additional questions after 1 year and 4 years

One and 4 years after ICU discharge, most survivors lived independently, and only a minority stayed in a special care facility (Table 1). There were no major sleeping problems. One year and 4 years after ICU discharge, AKI-RRT patients had more bad memories than non-AKI-RRT patients (after 1 year, 17.4 % versus 4.3 %, *P* = 0.010; after 4 years, 21.4 % versus 3.8 %, *P* = 0.055). Of the 1-year AKI-RRT patients 81.8 % preferred to be readmitted to an ICU department in case of deterioration versus 83.0 % of their 1-year matches (*P* = 0.867). This number decreased to 71.4 % for the 4-year AKI-RRT patients versus 84.6 % for the 4-year non-AKI-RRT patients (*P* = 0.244).

## Discussion

In this prospective, single-center matched cohort study concerning long-term outcomes and QOL of AKI-RRT patients, we found high mortality rates and lower QOL levels compared to the general population.

Similar to others, we found high hospital mortality (55 %) in this cohort of critically ill AKI-RRT patients, with only moderate increases in mortality at longer follow-up (58 % at 3 months, 61 % at 1 year, 71 % at 4 years) [4, 14, 15, 20, 36].

At hospital discharge and at long term, a quarter of AKI-RRT hospital survivors were RRT-dependent. These findings are similar to those reported in the literature [37].

Long-term survival data would be meaningless without considering QOL. Remarkably, there was no difference in QOL at different time points between AKI-RRT patients and matched non-AKI-RRT patients, although changes in QOL over time were less pronounced in the latter group. QOL decreased 3 months after ICU discharge compared to baseline, improved after 1 year, and stayed the same or improved slightly after 4 years, but still remained under baseline level.

The fact that long-term QOL had the same evolution over time in AKI-RRT and non-AKI-RRT patients was quite surprising suggesting that the AKI-RRT component during critical illness did not have an important impact on long-term QOL. Others reported very similar findings; however, these studies reported only on QOL after 6 months, and in one study not all AKI patients received RRT, and some patients received RRT without AKI [20, 21].

The fact that AKI-RRT patients were more severely ill during their ICU stay compared to matched patients had no influence on QOL over the years. This is in accordance with the findings of Orwelius et al. [38]. In a multicenter study they found that, 6 months after ICU discharge, perceived QOL in sepsis patients did not differ from ICU survivors with other diagnoses, even though these sepsis patients were more severely ill, and had a longer ICU stay. Another study by Orwelius suggested that long-term QOL was mainly affected by co-morbidity [39]. In our study AKI-RRT and non-AKI-RRT patients had a very comparable co-morbidity and medical history, which may explain the comparable long-term QOL between groups in our study.

QOL was perceived as acceptable and both AKI-RRT and non-AKI-RRT patients reported low dependence in daily life later on. The number of AKI-RRT and non-AKI-RRT patients who agreed to undergo life-sustaining interventions again in case of deterioration remained high. However, QOL was lower compared to that of the average population in both groups specifically in the more physical domains. This is in accordance with the findings of others [12–16, 20, 21].

Our study has several strengths. First, the matched cohort design demonstrates the real impact of AKI-RRT upon long-term QOL. This has not been evaluated thus far. Second, QOL was assessed with validated questionnaires at baseline, which allows for the only reliable evaluation of QOL over time without recall or selection bias [11, 40]. Third, the additional questions and VAS score allowed evaluation of the patients’ perception of the ICU admission and the consequences of severe illness. Finally, most studies report QOL in AKI survivors as a short-term endpoint, while this study also provides data for a longer follow-up period. Strict time intervals of 3 months and 1 year after ICU discharge were respected in all patients. For long-term assessment of QOL, an arbitrary time point was chosen (August 2013) which was between 47 and 52 months after ICU discharge for all patients. Response rate was very high and only three patients were lost to follow-up.

Some limitations should also be mentioned. First, single-center data from a university hospital may not reflect general practice and may limit external validity of the data. Second, although 1-year and 4-year AKI-RRT patients were matched to non-AKI-RRT patients based on four criteria, we cannot exclude that matched patients had a different profile compared to AKI-RRT patients. Third, the study cohort is relatively small and may lack statistical power to detect differences among the QOL domains in our study patients. Fourth, medical decisions leading to ICU referral may have selected for patients with better prospects. Fifth, long-term QOL may also be modified by events happening to the patient after hospital discharge. These were not recorded in the present study.

## Conclusions

We found high mortality rates in AKI-RRT patients. However, in long-term critically ill AKI-RRT survivors, QOL was comparable to matched long-term critically ill survivors without AKI-RRT, but lower than in the general population. The majority of AKI-RRT patients wanted to be readmitted to the ICU when needed, despite a higher severity of illness compared to matched non-AKI-RRT patients, and despite the fact that one quarter had persistent dialysis dependency.

## Key messages

- Long-term critically ill AKI-RRT survivors have comparable QOL to matched long-term critically ill survivors without RRT.
- QOL in long-term AKI-RRT survivors is lower than in the general population.
- AKI-RRT patients are more severely ill during their ICU stay compared to matched non-AKI-RRT patients.
- The majority of long-term AKI-RRT survivors prefer to be readmitted to the ICU department in case of deterioration.
- One quarter of long-term AKI-RRT survivors have persistent dialysis dependency.

## Additional files

Additional file 1:
**EQ-5D assessments over time. **In this additional file, evolutions in EQ-5D assessments are described through figures in the 1-year cohort (47 AKI-RRT (A) and 94 non-AKI-RRT patients (B)) and in the 4-year cohort (28 AKI-RRT (C) patients and 28 non-AKI-RRT patients (D)). Percentages of patients with some or severe problems in the different dimensions of the EQ-5D are given over the different time points: baseline, 3 months and 1 year (1-year cohort) and baseline, 3 months, 1 year and 4 years (4-year cohort). (PDF 111 kb) Additional file 2:
**SF-36 assessments over time.** In this additional file, evolutions in SF-36 assessments are described through figures in the 1-year cohort (47 AKI-RRT (A) and 94 non-AKI-RRT patients (B)) and in the 4-year cohort (28 AKI-RRT (C) patients and 28 non-AKI-RRT patients (D)). Percentages of patients with some or severe problems in the different domains of the SF-36 are given over the different time points: baseline, 3 months and 1 year (1-year cohort) and baseline, 3 months, 1 year and 4 years (4-year cohort). (PDF 126 kb) Additional file 3:
**Variability in EQ-5D.** In this additional file, more detailed information is given regarding variability of the EQ-5D at the different time points in the 1-year cohort and 4-year cohort. Percentages and 95 % confidence intervals of patients with some or severe problems on the respective dimensions of the EQ-5D over time are given in a table. (PDF 60 kb) Additional file 4:
**Variability in SF-36.** In this additional file, more detailed information is given regarding variability of the SF-36 at the different time points in the 1-year cohort and 4-year cohort. Median norm-based scores with interquartile ranges on the different domains of the SF-36 over time are given in a table. (PDF 81 kb)

## Abbreviations

AKI: Acute kidney injury
APACHE: Acute Physiology and Chronic Health Evaluation
BP: Bodily pain
eGFR: Estimated glomerular filtration rate
EQ-5D: EuroQoL-5D
GH: General health
ICU: Intensive care unit
IQR: Interquartile range
LOS: Length of stay
MCS: Mental component score
MH: Mental health
MICU: Medical intensive care unit
PCS: Physical component score
PF: Physical functioning
QOL: Quality of life
RE: Role emotional
RP: Role physical
RRT: Renal replacement therapy
SF: Social functioning
SF-36: Medical Outcomes Study 36-item Short Form Health Survey
SICU: Surgical intensive care unit
SOFA: Sequential Organ Failure Assessment
VAS: Visual analogue scale
VT: Vitality
