# Supplementary material for: Long-term quality of life in critically ill patients with acute kidney injury treated with renal replacement therapy: a matched cohort study
Source: Crit Care. 2015 Aug 6;19(1):289. doi: 10.1186/s13054-015-1004-8 (PMC4527359; doi:10.1186/s13054-015-1004-8)
Supplement: Additional file 1: — EQ-5D assessments over time. In this additional file, evolutions in EQ-5D assessments are described through figures in the 1-year cohort (47 AKI-RRT (A) and 94 non-AKI-RRT patients (B)) and in the 4-year cohort (28 AKI-RRT (C) patients and 28 non-AKI-RRT patients (D)). Percentages of patients with some or severe problems in the different dimensions of the EQ-5D are given over the different time points: baseline, 3 months and 1 year (1-year cohort) and baseline, 3 months, 1 year and 4 years (4-year cohort). (PDF 111 kb) [file 13054_2015_1004_MOESM1_ESM.pdf]

Additional File 1: EQ-5D assessments over time

Percentages of patients with some or severe problems per dimension

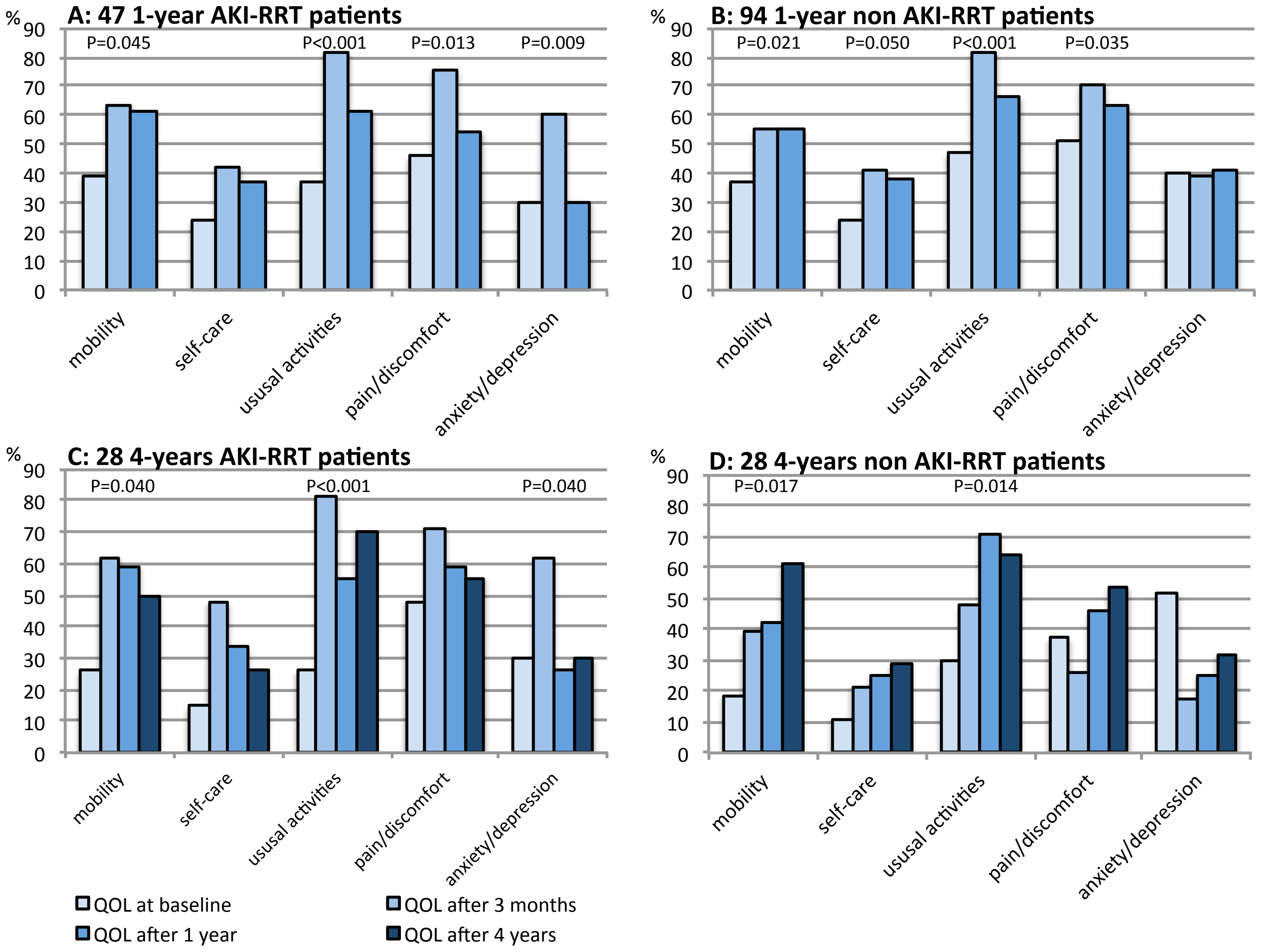

The X-axis represents the different dimensions of the EQ-5D.

The Y-axis represents the percentages (%) of patients with some or severe problems in a respective dimension.

Only significant P-values (Chi Square test) are shown above the respective dimensions.

<u>Abbreviations</u>: QOL= quality of life; AKI= acute kidney injury; RRT= renal replacement therapy
