# Supplementary material for: Long-term quality of life in critically ill patients with acute kidney injury treated with renal replacement therapy: a matched cohort study
Source: Crit Care. 2015 Aug 6;19(1):289. doi: 10.1186/s13054-015-1004-8 (PMC4527359; doi:10.1186/s13054-015-1004-8)
Supplement: Additional file 2: — SF-36 assessments over time. In this additional file, evolutions in SF-36 assessments are described through figures in the 1-year cohort (47 AKI-RRT (A) and 94 non-AKI-RRT patients (B)) and in the 4-year cohort (28 AKI-RRT (C) patients and 28 non-AKI-RRT patients (D)). Percentages of patients with some or severe problems in the different domains of the SF-36 are given over the different time points: baseline, 3 months and 1 year (1-year cohort) and baseline, 3 months, 1 year and 4 years (4-year cohort). (PDF 126 kb) [file 13054_2015_1004_MOESM2_ESM.pdf]

Additional File 2: SF-36 assessments over time: Norm-based median per domain

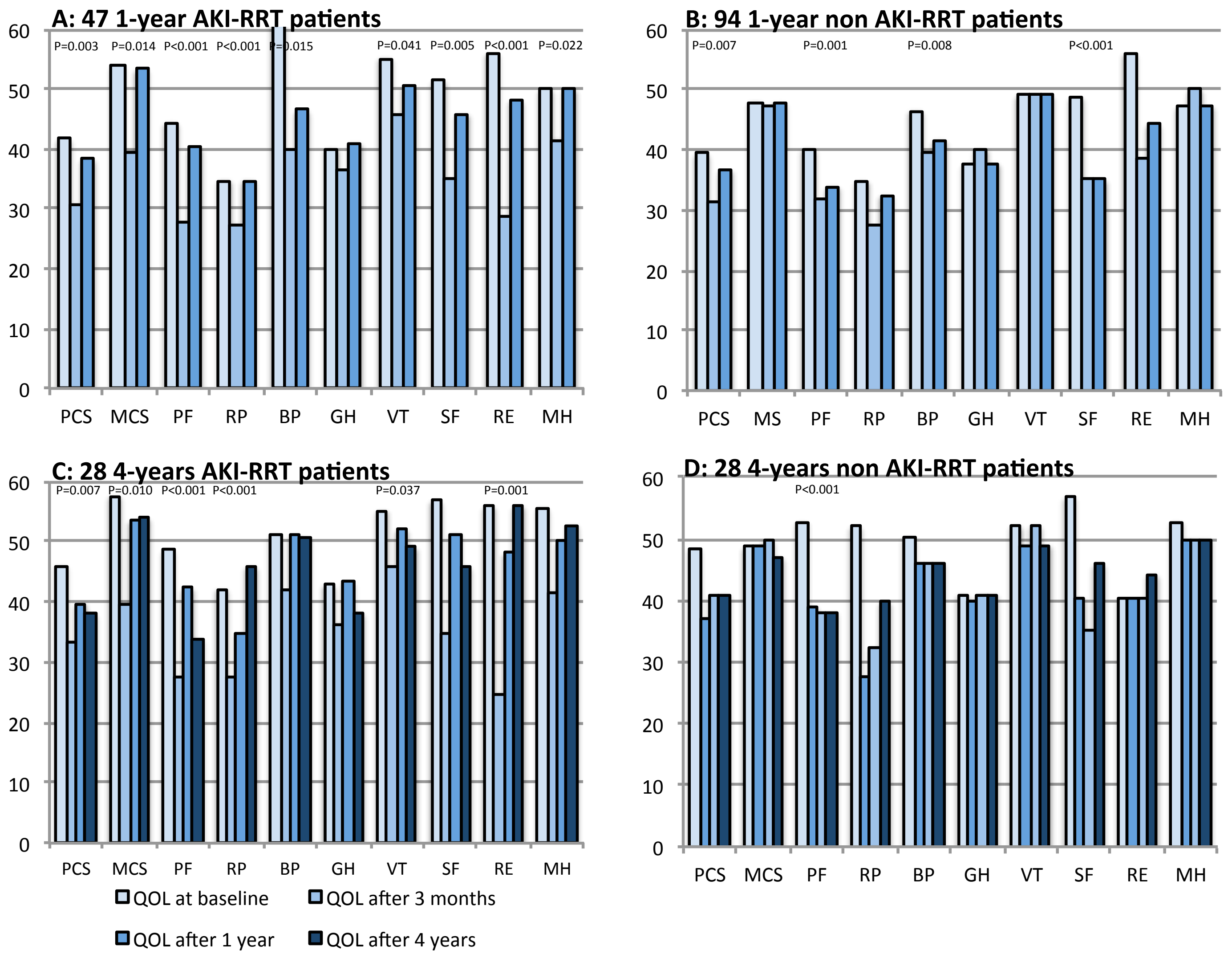

The X-axis represents the different domains of the SF-36.

The Y-axis represents the norm-based median scores in a respective domain of the SF-36. A norm-based median score between 47-53 in a group of patients is considered as normal or average. Norm-based median scores below 47 indicate impaired functioning or below average; norm-based median scores above 53 indicate better functioning or above average.

Only significant P-values (Friedman test) are shown above the respective domains.

Abbreviations: **QOL= quality of life; AKI= acute kidney injury; RRT= renal replacement therapy PCS= physical component score; MCS= mental component score; PF= physical functioning; RP= role physical; BP = bodily pain; GH= general health; VT= vitality; SF= social functioning; RE= role emotional; MH= mental health**
