# Supplementary material for: Long-term quality of life in critically ill patients with acute kidney injury treated with renal replacement therapy: a matched cohort study
Source: Crit Care. 2015 Aug 6;19(1):289. doi: 10.1186/s13054-015-1004-8 (PMC4527359; doi:10.1186/s13054-015-1004-8)
Supplement: Additional file 3: — Variability in EQ-5D. In this additional file, more detailed information is given regarding variability of the EQ-5D at the different time points in the 1-year cohort and 4-year cohort. Percentages and 95 % confidence intervals of patients with some or severe problems on the respective dimensions of the EQ-5D over time are given in a table. (PDF 60 kb) [file 13054_2015_1004_MOESM3_ESM.pdf]

Additional File 3: Variability of the EQ-5D at the different time points: Percentages and 95% confidence intervals (*) of patients with some or severe problems on the respective dimensions

**47 1-year AKI-RRT patients**					
	**Baseline**	**3 months**	**1 year**	**P**	
% (95% CI)					
Mobility	39.1 (26.4-53.5)	63.6 (46.6-77.8)	60.9 (46.5-73.6)	0.045	
Self-care	23.9 (13.9-37.9)	42.4 (27.2-59.2)	37.0 (24.5-51.4)	0.190	
Ususal activities	37.0 (24.5-51.4)	81.8 (65.6-91.4)	60.9 (46.5-73.6)	<0.001	
Pain/discomfort	45.7 (32.2-59.8)	75.8 (59.0-87.2)	54.3 (40.2-67.8)	0.013	
Anxiety/depression	30.4 (19.1-44.8)	60.6 (43.7-75.3)	30.4 (19.1-44.8)	0.009	
**94 1-year non AKI-RRT patients**					
	**Baseline**	**3 months**	**1 year**	**P**	
% (95% CI)					
Mobility	37.2 (28.1-47.3)	54.9 (43.4-66.0)	55.4 (45.3-65.2)	0.021	
Self-care	24.5 (16.9-34.0)	40.8 (30.2-52.5)	38.0 (28.8-48.3)	0.050	
Ususal activities	46.8 (37.0-56.8)	81.7 (71.2-89.0)	66.3 (56.2-75.1)	<0.001	
Pain/discomfort	51.1 (41.1-60.9)	70.4 (59.0-79.8)	63.0 (52.8-72.2)	0.035	
Anxiety/depression	40.4 (31.1-50.5)	39.4 (28.9-51.1)	41.3 (31.8-51.5)	0.971	
**28 4-years AKI-RRT patients**					
	**Baseline**	**3 months**	**1 year**	**4 years**	**P**
% (95% CI)					
Mobility	25.9 (13.2-44.7)	61.9 (40.9-79.2)	59.3 (40.7-75.5)	50.0 (32.6-67.4)	0.040
Self-care	14.8 (5.9-32.5)	47.6 (28.3-67.6)	33.3 (18.6-52.2)	25.9 (13.2-44.7)	0.090
Ususal activities	25.9 (13.2-44.7)	81.0 (60.0-92.3)	55.6 (37.3-72.4)	70.4 (51.5-84.1)	<0.001
Pain/discomfort	48.1 (30.7-66.0)	71.4 (50.0-86.2)	59.3 (40.7-75.5)	55.6 (37.3-72.4)	0.439
Anxiety/depression	29.6 (15.9-48.5)	61.9 (40.9-79.2)	25.9 (13.2-44.7)	29.6 (15.9-48.5)	0.040

28 4-years non AKI-RRT patients					
	**Baseline**	**3 months**	**1 year**	**4 years**	**P**
% (95% CI)					
Mobility	18.5 (8.2-36.7)	39.1 (22.2-59.2)	41.7 (24.5-61.2)	60.7 (42.4-76.4)	0.017
Self-care	11.1 (3.9-28.1)	21.7 (9.7-41.9)	25.0 (12.0-44.9)	28.6 (15.3-47.1)	0.436
Ususal activities	29.6 (15.9-48.5)	47.8 (29.2-67.0)	70.8 (50.8-85.1)	64.3 (45.8-79.3)	0.014
Pain/discomfort	37.0 (21.5-55.8)	26.1 (12.5-46.5)	45.8 (27.9-64.9)	53.6 (35.8-70.5)	0.227
Anxiety/depression	51.9 (34.0-69.3)	17.4 (7.0-37.1)	25.0 (12.0-44.9)	32.1 (17.9-50.7)	0.054

Abbreviations: AKI= acute kidney injury; RRT= renal replacement therapy; CI=confidence interval

(*) The confidence interval was calculated according to DG Altman, D Machin,TN Bryant, M Gardner (2000). Statistics with confidence: Confidence intervals and statistical guidelines. BMJ Books
