# Supplementary material for: Long-term quality of life in critically ill patients with acute kidney injury treated with renal replacement therapy: a matched cohort study
Source: Crit Care. 2015 Aug 6;19(1):289. doi: 10.1186/s13054-015-1004-8 (PMC4527359; doi:10.1186/s13054-015-1004-8)
Supplement: Additional file 4: — Variability in SF-36. In this additional file, more detailed information is given regarding variability of the SF-36 at the different time points in the 1-year cohort and 4-year cohort. Median norm-based scores with interquartile ranges on the different domains of the SF-36 over time are given in a table. (PDF 81 kb) [file 13054_2015_1004_MOESM4_ESM.pdf]

Additional File 4: Variability of the SF-36 norm-based scores at the different time points: median and interquartile ranges

**47 1-year AKI-RRT patients**				
	**Baseline**	**3 months**	**1 year**	**P**
Median (IQR)				
PCS	41.7 (28.5-54.2)	30.7 (25.1-40.4)	38.3 (27.7-47.4)	0.003
MCS	53.8 (38.9-61.6)	39.5 (29.3-47.2)	53.3 (39.2-58.6)	0.014
Physical functioning	44.4 (29.1-53.4)	27.6 (19.2-39.1)	40.2 (26.5-46.5)	<0.001
Role physical	34.8 (22.6-56.9)	27.5 (17.7-29.9)	34.8 (25.0-45.8)	<0.001
Bodily pain	62.1 (37.2-62.1)	39.7 (29.2-50.9)	46.5 (37.2-62.1)	0.015
General health	40.1 (30.5-48.2)	36.3 (31.1-41.0)	41.0 (30.5-50.6)	0.078
Vitality	55.2 (42.7-61.5)	45.8 (39.6-50.5)	50.5 (41.9-59.1)	0.041
Social functioning	51.4 (35.0-56.8)	35.0 (24.1-40.5)	45.9 (29.6-56.8)	0.005
Role emotional	55.9 (40.3-55.9)	28.7 (20.9-38.4)	48.1 (32.6-55.9)	<0.001
Mental health	50.0 (33.1-61.3)	41.6 (30.3-50.0)	50.0 (40.2-58.4)	0.022
**94 1-year non AKI-RRT patients**				
	**Baseline**	**3 months**	**1 year**	**P**
Median (IQR)				
PCS	39.4 (29.1-49.6)	31.3 (26.3-43.2)	36.6 (26.0-46.4)	0.007
MCS	48.0 (37.5-55.7)	47.3 (31.6-54.9)	47.8 (34.8-54.0)	0.759
Physical functioning	40.2 (23.4-53.4)	31.8 (21.3-44.4)	33.9 (22.3-48.6)	0.001
Role physical	34.8 (22.6-56.9)	27.5 (17.7-37.3)	32.4 (23.2-42.2)	0.059
Bodily pain	46.5 (33.3-62.1)	39.5 (29.2-50.5)	41.6 (29.2-55.4)	0.008
General health	37.7 (30.5-50.6)	40.1 (31.1-45.8)	37.7 (30.5-45.8)	0.871
Vitality	49.0 (36.5-58.3)	49.0 (39.6-55.2)	49.0 (36.5-58.3)	0.896
Social functioning	48.7 (35.0-56.8)	35.0 (24.1-45.9)	35.0 (24.1-51.4)	<0.001
Role emotional	55.9 (31.6-55.9)	38.4 (20.9-55.9)	44.2 (24.8-55.9)	0.410
Mental health	47.2 (33.1-58.4)	50.0 (34.5-55.7)	47.2 (34.5-55.6)	0.562

**28 4-years AKI-RRT patients**					
	**Baseline**	**3 months**	**1 year**	**4 years**	**P**
Median (IQR)					
PCS	46.1 (38.7-53.7)	33.2 (26.0-40.4)	39.8 (31.6-46.7)	38.1 (31.6-47.1)	0.007
MCS	57.6 (42.8-62.3)	39.5 (29.3-47.1)	53.5 (40.9-61.6)	53.9 (42.4-60.3)	0.010
Physical functioning	48.6 (36.5-57.0)	27.6 (18.1-43.4)	42.3 (29.7-48.6)	33.9 (29.7-40.2)	<0.001
Role physical	42.2 (27.5-56.9)	27.5 (17.7-31.8)	34.8 (27.5-47.1)	45.9 (27.5-56.9)	<0.001
Bodily pain	51.1 (38.2-62.1)	41.8 (30.1-50.9)	51.1 (41.8-62.1)	50.7 (34.4-62.1)	0.178
General health	42.9 (30.3-47.9)	36.3 (32.9-42.9)	43.4 (36.3-50.6)	38.2 (32.9-48.0)	0.093
Vitality	55.2 (43.5-64.6)	45.8 (42.7-50.5)	52.1 (45.8-61.5)	49.0 (45.8-58.3)	0.037
Social functioning	56.8 (40.5-56.8)	35.0 (26.9-40.5)	51.4 (35.0-56.8)	45.9 (35.0-56.8)	0.101
Role emotional	55.9 (50.0-55.9)	24.8 (9.2-38.4)	48.1 (32.6-55.9)	55.9 (20.9-55.9)	0.001
Mental health	55.6 (33.1-64.1)	41.6 (33.1-51.4)	50.0 (41.6-61.3)	52.8 (41.6-58.5)	0.188
**28 4-years non AKI-RRT patients**					
	**Baseline**	**3 months**	**1 year**	**4 years**	**P**
Median (IQR)					
PCS	48.4 (36.3-57.0)	37.1 (26.1-45.5)	40.8 (27.9-46.5)	41.0 (32.1-52.6)	0.358
MCS	48.6 (34.3-57.6)	48.9 (37.2-54.8)	49.7 (40.6-54.7)	47.0 (37.4-55.5)	0.913
Physical functioning	52.8 (40.2-54.9)	39.1 (19.2-44.4)	38.1 (22.3-48.6)	38.1 (25.5-48.6)	<0.001
Role physical	52.0 (17.7-56.9)	27.5 (25.0-39.7)	32.4 (25.0-39.7)	39.7 (25.0-47.1)	0.158
Bodily pain	50.3 (41.2-62.1)	46.1 (37.2-55.4)	46.1 (36.1-62.1)	46.1 (37.2-62.1)	0.489
General health	41.0 (35.3-55.3)	40.1 (29.8-49.4)	41.0 (35.3-48.8)	41.0 (34.7-53.5)	0.577
Vitality	52.1 (42.7-58.3)	49.0 (39.6-58.3)	52.1 (39.6-58.3)	49.0 (42.7-55.2)	0.403
Social functioning	56.8 (35.0-56.8)	40.5 (24.1-51.4)	35.0 (22.8-52.8)	45.9 (24.1-56.8)	0.058
Role emotional	40.3 (20.9-55.9)	40.3 (28.7-55.9)	40.3 (24.8-55.9)	44.2 (24.8-55.9)	0.071
Mental health	52.8 (35.9-58.4)	50.0 (37.3-58.5)	50.0 (37.3-58.5)	50.0 (41.6-52.8)	0.962

Abbreviations: AKI= acute kidney injury; RRT= renal replacement therapy; IQR= interquartile range (25%-75%); PCS= physical component score; MCS= mental component score
